# Impact of Sodium-Glucose Co-Transporter Type-2 Inhibitors on Alanine Aminotransferase Levels in Type-2 Diabetes Patients Having Features of Nonalcoholic Fatty Liver Disease: A Retrospective Cohort Study in Pakistan

**DOI:** 10.12669/pjms.40.11.8900

**Published:** 2024-12

**Authors:** Asefa S. Ansari, Azra Rizwan, Uzma Z. Khan, Syed Iqbal Azam

**Affiliations:** 1 Asefa S.Ansari, MSc Research Consultant, Department of Cardiology Research, Tabba Heart Institute, Karachi, Pakistan. Former Lecturer, Department of Public Health, Shaheed Zulfikar Ali Bhutto Institute of Science and Technology, (SZABIST) University., Aga Khan University Hospital (AKUH), Karachi, Pakistan; 2 Azra Rizwan, FCPS Consultant Diabetes and Endocrinology, Aga Khan University Hospital (AKUH), Karachi, Pakistan; 3 Uzma Z.Khan, MD Fellow, Division of Endocrinology, University of Mississippi Medical Center, Jackson Mississippi, Professor of Medicine, Cosmopolitan International Diabetes and Endocrinology Center, University of Missouri-Columbia, One Hospital Drive, Columbia, MO 65212; 4 Mr. Syed Iqbal Azam, BSc Assistant Professor, Department of Community Health Sciences, Aga Khan University Hospital (AKUH), Karachi, Pakistan

**Keywords:** Sodium-Glucose Cotransporter-2 inhibitors (SGLT2-I), Non-alcoholic Fatty Liver Disease (NAFLD), Type 2 Diabetes Mellitus, Fatty liver disease, Alanine Amino-transaminase

## Abstract

**Objective:**

We aimed to elucidate the effectiveness of Sodium-glucose co-transporter-2 inhibitors (SGLT2-I) in the reduction of ALT among Type-2 diabetes patients (T2DM) with Non-alcoholic fatty liver disease (NAFLD).

**Methods:**

We retrospectively collected data from 120 files of T2DM, aged 30-60 years, with elevated ALT, and documented follow-up for one year from August 2018 - July 2019. The effects of SGLT2Is (Dapagliflozin and Empagliflozin) were evaluated using Generalized Estimating Equation (GEE) for analysis.

**Results:**

The overall mean age was 48.9 ± 7.3 years, 57.5% were females, and the mean duration of diabetes was 8.5 ± 5.6 years. At baseline, the mean BMI was 32.5 ± 5.7 kg/m^2^, mean ALT was 51.6 IU/L ± 17.8 IU/L, and mean HbA1c was 8.5% ± 1.5%. There was a statistically significant reduction in mean ALT of 2.2 IU/L (p-value 0.02) with every 10 mg/dl increase in LDL among females using 10 mg Empagliflozin as compared to males not on SGLT2i.

**Conclusions:**

We observed an average reduction in mean ALT levels when SGLT2Is was initiated in T2DM patients having NAFLD. Apart from encouraging diet and lifestyle modification, early intervention with SGLT2Is may decrease liver-related morbidity and mortality resulting from NAFLD.

## INTRODUCTION

Nonalcoholic fatty liver disease (NAFLD) is a condition in which there is an accumulation of excess fat (steatosis) affecting at least 5% of liver tissue^1^ in people not known to have excessive alcohol exposure (drink alcohol < 20 g/day in male; < 10 g/day in female)^2^ The global estimated prevalence of NAFLD in patients with type 2 Diabetes Mellitus (T2DM) is estimated to be 55.48 (95% CI: 47.26-63.67).^3^ In Western countries, prevalence is reported to be nearly 70%^4^ in contrast to South Asia, where NAFLD prevalence varies from 9% to 45%.^5^-^7^ In Pakistan, a study conducted in a tertiary care hospital reported that 72.4% of patients with T2DM and metabolic syndrome had NAFLD.^8^ Liver diseases, such as liver cirrhosis and hepatocellular carcinoma, are among the leading causes of morbidity and mortality in T2DM patients.^9^-^11^

There is increasing evidence that T2DM subjects have a greater risk of developing NAFLD even in patients with normal Alanine Amino-transaminase (ALT levels).^11^ To date, there are no Food and Drug Administration (FDA) approved pharmacotherapies available locally for the treatment of NAFLD. Management strategies include diet modification and exercise of at least 150 mins/week, and treatment of comorbidities^12^ with an aim to reduce the ALT levels which may slow the progression of liver-related morbidity and mortality.

Sodium glucose co-transporter type 2 inhibitors (SGLT2-I) are FDA-approved oral anti-diabetic agents for the treatment of T2DM which exert their action by inhibiting glucose reabsorption via SGLT2 receptors in proximal tubules of kidneys.^13^ The availability of SGLT2Is in oral forms is both convenient and cost-effective. SGLT2Is exert cardio-protective and reno-protective actions as well as have beneficial effects on metabolic parameters, including ALT levels.^14^ Significant improvements in liver enzymes was observed among Canagliflozin users as compared to placebo and Sitagliptin groups, slowing the progression of fatty liver in patients with T2DM.^15^ Local data regarding the effects of SGLT2Is on ALT levels are scarce; therefore, this study will provide evidence to assess their benefits in Pakistan. We aimed to evaluate the changes in ALT levels during a period of one year after initiation of SGLT2Is in T2DM patients, aged 30 - 60 years, having NAFLD, presenting to an Endocrine outpatient clinic of tertiary care hospital in Karachi.

## METHODS

This was a retrospective, longitudinal, single-centered cohort study over a period of one year, to evaluate the effects of two different SGLT2-I, i.e., Dapagliflozin (5 & 10 mg) and Empagliflozin (10 mg, 12.5 mg & 25 mg), when included in the standard treatment of patients with T2DM having NAFLD. This study was a part of thesis project conducted during the COVID-19 pandemic. The study design did not have a control group, since the data, results, and measurements collected were part of routine diabetes care, with most of the subjects prescribed SGLT2Is during the defined study period.

### Study Population:

Files of all patients presenting to the Agha Khan University Hospital outpatient clinics, with uncontrolled T2DM (HbA1c ≥ 7%), aged 30 – 60 years, having ALT levels ≥ 40 IU/L for males and ≥ 30 IU/L for females, who had been initiated on oral SGLT2-I, and had followed up for at-most three visits during the period of August 2018 - July 2020 were reviewed.

Exclusion criteria included patients with conditions known to affect liver enzymes, such as hepatitis B, hepatitis C,^16^ autoimmune hepatitis,^1^,^16^-^17^ drugs induced liver damage,^17^ alcohol intake of 10 - 30g/day (>2 drinks/day for females and >3drinks/day for males) within the previous year,^16^ and the recent initiation (within 12 weeks) of medications that cause hepatic steatosis.^16^ Patients with known cirrhosis, or hepatocellular carcinoma on U/S or MRI, pregnant women, renal insufficiency [eGFR ≤ 45 mL/min/1.73 m^2^],^18^ and severely ill patients (history of high-grade fever, sepsis or acute infection) or patients with ALT value above three times the upper limit of normal range^19^ were also excluded.

As this was a retrospective study, patients’ autonomy was not affected, and no informed consent was required. The Study Questionnaire was adapted from a validated Questionnaire tool, The Diabetes Self-Management Questionnaire (DSMQ), having Cronbach’s alpha of 0.84.^20^

### Ethical Approval:

This study was approved by the Ethical Review Committee (ERC) of Aga Khan University Hospital [ERC Reference Number: 3522].

### Statistical Analysis:

A sample size of 184 T2DM comorbid with NAFLD was calculated to achieve a power of 80% with a two-sided level of significance of 5%, anticipated mean paired difference of 45.0 IU/L or more, anticipated standard deviations (SD) of pre and post treatment ALT level of 115 IU/L and 78 IU/L respectively, with a correlation co-efficient of two readings of ALT of 0.024, changes in ALT observed four times at most, with a drop rate of <10%.^21^ NCSS PASS software was used to estimate the sample size. The target sample size was not achieved due to the COVID-19 pandemic during the designated study period.

Purposive sampling was used to select files from the Diabetes Database and Health Information and Management System (HIMS). Rigorous application of eligibility criteria reduced selection biases due to non-random sampling techniques. Continuous variables were checked for normality by One-sample Kolmogorov-Smirnov test and reported as means with standard deviations, whereas categorical variables were reported as proportions and frequencies. We observed approximately 30% of missing data related to various variables. Imputation for missing data related to our independent variables, utilized Last Observation Carried Forward (LOCF) method for fasting blood sugar (FBS), random blood sugar (RBS), glycosylated hemoglobin A1c (HbA1c), and Multiple Imputation analyses for ALT, low-density lipoprotein cholesterol (LDL), high-density lipoprotein cholesterol (HDL) and triglycerides (TG)], followed by re-calculation of the means with standard deviations for these variables.

Two-way scatter plots were plotted to observe the relationship between independent continuous variables and changes in ALT level. Variables that were significant in univariate analysis (having p-value <0.25), were checked for multi-collinearity by performing Pearson’s correlation coefficient, eta and cramer. Generalized estimating equation (GEE) for multivariable analysis was then applied, using an unstructured covariance structure and Stepwise selection method to construct the model. Overall, level of significance was taken as p-value of less than 0.05 and was based on two-sided tests. Interaction [p-value <0.1] between biologically plausible variables, such as age, gender, BMI, and glycemic parameters was assessed. STATA 14.2 software (STATA Corp, College Station, Texas, USA) was used to perform all statistical analysis.

### Clinical and Laboratory Data:

Data was collected to examine changes in study parameters at the initiation of study drug (baseline), and at three, six and twelve months subsequently through data collected from Diabetic forms of patients’ files. The primary outcome variable was change in ALT level (normal range for ALT is 7-45 IU/L for males and 7-35 IU/L for females). The primary exposure was the introduction of an SGLT2Is (as single or combination forms). Patient factors included age, sex, marital status, diet control, and physical activity. Normal BP value was considered as 120/80 mmHg. Body mass index (BMI) was calculated as weight(kg) divided by height squared (m^2^), and normal range of BMI among Asians was taken as 18-23 kg/m^2^.^22^ Information regarding adherence to study drug, adverse effects of SGLT2Is and other medications taken by patients was obtained. Laboratory data, including FBS, RBS, HbA1c, and lipid profile [including LDL, HDL and TG] and liver function tests (LFTs) were collected.

## RESULTS

### Patient Characteristics:

The overall mean age of 120 patients with T2DM and elevated ALT was 48.9 ± 7.3 years, and mean duration of diabetes was found to be 8.5 ± 5.6 years. 57.5% were females. Maximum follow-up was observed at 6 months [91.7%], whereas only 77.5% of patients followed up at 12 months. The baseline characteristics distribution of the sample population are shown in Table-I. At baseline, the mean BMI was found to be 32.5 ± 5.7 kg/m^2^. 43.5% of females were obese with BMI above 35 kg/m^2^, whereas, 49% of males were between 30 and 35 kg/m^2^. A non-significant reduction of 0.79 kg/m^2^ was observed in males as compared to 1.49 kg/m^2^ in females during 12 months’ follow-up. 42.5% [n=51] of patients had NAFLD detected in ultrasound (US) based on at least ≥30% hepatic steatosis with other features of fatty liver.

**Table-I:** Patients’ socio-demographic and health related characteristics distribution at baseline (n=120).

Variables	Frequency (Percent)
Age Groups (years)*	
30 - 39	18 (15.0)
40 - 49	43 (35.8)
50 - 60	59 (49.2)
Height (cm)*	
Mean (SD)	162.2 (9.2)
Religion*	
Islam	115 (95.8)
Christian	01 (0.8)
Hindu	04 (3.3)
***Ethnicity^†^***	
Urdu	59 (49.2)
Sindhi	09 (7.5)
Balochi	02 (1.7)
Pashto	06 (5.0)
Memon	01 (0.8)
Afghani	01 (0.8)
Not specified	42 (35)
***Place of Residence^‡^***	
Karachi	65 (54.2)
Other than Karachi	28 (23.3)
Not specified	27 (22.5)
***Occupation^§^***	
Retired	01 (0.008)
House wife	38 (31.7)
Employed Not specified	25 (20.8) 56 (46.7)
Follows Diet instructions*	
Always	25 (20.8)
Sometimes	84 (70.0)
Never	11 (9.2)
Physical activity reported*	
Always	23 (19.2)
Sometimes	32 (26.7)
Never	65 (54.2)
Medicine adherence*	
Always	100 (83.3)
Sometimes	20 (16.7)
Never	0

* n = 120; † n = 78; ‡ n = 90; § n = 64; SD: standard deviation.

### Changes in Study Parameters:

**Fig.1-A:**
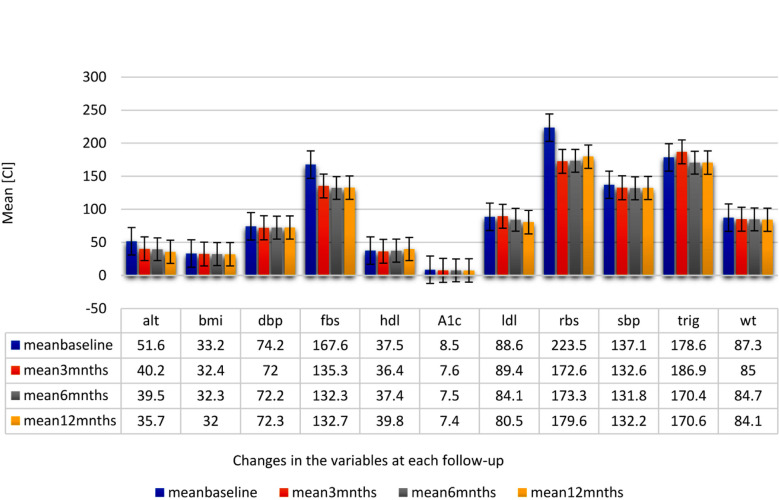
Legend: Changes in the mean (95% CI) of patients’ metabolic and hemodynamic factors from baseline visit (blue-colored) till 12 months. 3-month follow-up is represented by orange color, 6-month follow-up is represented by grey color, and 12-month follow-up is represented by yellow color. alt: Alanine Aminotransferase; bmi: Body Mass Index; dbp: Diastolic Blood Pressure, fbs: Fasting Blood Sugar; hdl: High Density Lipoprotein; A1c: Glycated Hemoglobin; ldl: Low Density Lipoprotein; rbs: Random Blood Sugar; sbp: Systolic Blood pressure; trig: Triglycerides; wt: weight; SGLT2-i: Sodium Glucose Cotransporter-2 inhibitors; CI: 95% confidence interval; I : standard error bars.

At baseline, mean ALT was 51.6 IU/L ± 17.8 IU/L, mean LDL was 88.6 ± 33.2 mg/dl, and mean HbA1c was 8.5% ± 1.5% (Fig.1-A). There was a decrease in mean ALT level of 11.5 IU/L at 3 months, 12.1 IU/L at 6 months and.15.9 IU/L at 12 months from baseline (Fig.1-A &1-B). Lipid profile showed an initial increase in mean LDL level from baseline and later a reduction in subsequent follow-ups (89.4 mg/dl, 84.1 mg/dl, and 80.5 mg/dl, respectively). The mean FBS, RBS, HbA1c, and other covariates (such as BW, BMI, SBP, DBP, TG, LDL, and HDL) showed a non-significant reduction (Fig.1-A). Approximately, 15% of the patients developed adverse events related to the study drug, most reporting increased frequency of urine (10.2%), with two patients reporting urinary tract infection.

**Fig.2-B:**
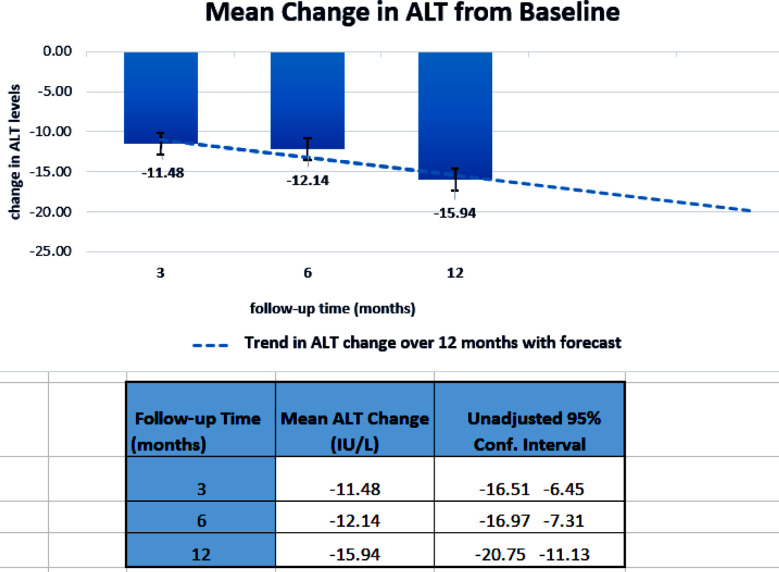
Legend: Mean change in ALT levels from baseline visit, to each follow-up at 3 months, 6 months, and 12 months (blue-colored boxes) subsequently. The dashed line shows the trend in ALT change observed over 12 months and forecasts the future of further reduction in ALT that might have occurred if the study was extended. ALT: Alanine Aminotransferase; 0.00: baseline mean ALT; I :standard error bars; Conf.Interval: 95% confidence interval.

Univariate analysis found a maximum reduction of 10.4 IU/L in ALT levels in the age group between 50-60 years (p-value 0.003, 95% CI -17.2 to -3.5). As compared to males, there was statistically significant reduction in mean ALT of 10.6 IU/L among females (p-value <0.0001, 95% CI -15.1 to -6.0). BMI as a continuous variable was found to be insignificant but as a categorical variable, there was a statistically significant increase in ALT observed in overweight and obese patients as compared to normal weight subjects (p-value 0.0001). A statistically significant increase in mean ALT was observed with an increase in HbA1c (p-value 0.0004), FBS (p-value 0.0004), RBS (p-value 0.0002), TG (p-value < 0.0001), and LDL levels (p-value 0.0008) in addition to a statistically significant decrease in mean ALT of 3.7 IU/L with 10 mg/dl increase in HDL (p-value 0.01). Among the group of medicines that the T2DM patients were using, only SGLT2Is showed a significant reduction in mean ALT (p-value < 0.0001) at univariate analysis. A comparison in the outcome between males and females showed a statistically significant reduction of 11 IU/L in mean ALT levels among females as compared to males, keeping BMI constant in the model.

Multivariable analysis, using males not treated with SGLT2Is as a reference, identified covariates that were significant in the final model (X^2^ 174.7, df = 20, p-value <0.0001) that included the primary exposure (SGLT2-I), BMI, gender, LDL and TG, with three-way interaction observed between SGLT2-I, LDL, and gender. Gender and LDL acted as effect modifiers and in their presence, the reduction in mean ALT by SGLT2Is use was observed to have different patterns [Table-II]. For example, females using 10 mg Empagliflozin had a statistically significant reduction in mean ALT of 2.2 IU/L (p-value 0.02) with every 10 mg/dl increase in LDL compared to males not on SGLT2-I.

**Table-II:** Multivariable analysis for mean ALT reduction (n=120).

Variables	Adjusted Beta-Coefficient (95% CI / p-value)
***No SGLT2Is at Baseline and Male gender (Ref.)***	
5 mg Dapa use	-8.3 (-20.5 to 3.9)†
10 mg Dapa use	-9.9 (-15.1 to -4.8)
10 mg Emp use	-8.7 (-12.9 to -4.4)†
25 mg Emp use	-14.7 (-22.7 to -6.6)†
***BMI (kg/m^2^)***	
<23 kg/m^2^ (Ref)	1
23-24.9 kg/m^2^	32.2 (19.4 to 44.9)
25-29.9 kg/m^2^	14.4 (1.1 to 27.8)
30-34.9 kg/m^2^	15.9 (2.8 to 29.0)
>35 kg/m^2^	16.1 (2.7 to 29.4)
Female gender (no SGLT2-I)	5.9 (-6.1 to 18.1)
***With every 1 mg/dl increase in LDL, when:***	
No SGLT2Is with female gender	-0.2 (-0.3 to -0.1)^‡^
5 mg Dapa use with female gender	-0.1 (-0.3 to 0.02)
5 mg Dapa use with male gender	-0.08 (-0.2 to 0.06)
10 mg Dapa use with female gender	-0.1 (-0.4 to 0.08)
10 mg Dapa use with male gender	-0.06 (-0.2 to 0.1)
10 mg Emp use with female gender	-0.2 (-0.4 to -0.03)^‡^
10 mg Emp use with male gender	-0.09 (-0.2 to 0.1)
25 mg Emp use with female gender	-0.2 (-0.5 to 0.04)
25 mg Emp use with male gender	-0.2 (-0.6 to 0.1)
Triglyceride (mg/dl)	0.02 (0.002 to 0.04)^†^
LDL (mg/dl)	0.2 (0.1 to 0.3)^†^
Constant	20.7 (4.17 to 37.2)

† p-value <0.05; ‡ p-value <0.1; SGLT2-I: Sodium Glucose Cotransporter-2 inhibitors; Dapa: Dapagliflozin; Emp: Empagliflozin; Cana: Canagliflozin; LDL: Low Density Lipoprotein; CI: Confidence Interval; Ref: Reference.

## DISCUSSION

This retrospective, cohort study showed subjects with raised ALT levels and uncontrolled T2DM initiated on SGLT2Is had a significant reduction of mean ALT levels and other study parameters over a period of 12 months. Gender and LDL were found to be effect modifiers for observed pattern differences for the primary exposure (SGLT2-I). This study was unable to reach the target population of 184 participants, with only 120 charts available for review during the predefined study period. However, it provides important information for an understudied population in Pakistan, paving the way for future studies.

The strength of this study is the accuracy of data which was collected at a reputable tertiary care hospital in Karachi. The relationship between SGLT2Is and deranged ALT levels was examined using GEE to account for intra-subject correlation of outcomes. Another strength is The Diabetes Self-Management Questionnaire (DSMQ), a validated Questionnaire tool used for data collection. The highest follow-up was seen at the six-month visit, with a decrease at the 12-months visit. A longer follow-up period may have provided better insight into patient adherence to follow-up care and changes in study parameters. The loss to follow-up rate observed in this study may improve in future studies with the availability of health cards, reducing the financial burden of diabetes treatment which comes from patients’ pockets for most people in Pakistan.

In the study, there was a reduction of mean ALT in females, when keeping BMI constant. This finding was contrary to a study that observed better ALT improvements among males on SGLT2Is.^23^ This difference may be due to the dissimilar ethnic origins or lifestyle patterns between the two populations. The study showed a non-significant improvement in glycemic parameters (HbA1c, FBS, and RBS), lipid levels (TG, LDL, and HDL), and hemodynamic parameters (SBP, DBP). Other studies have shown a significant reduction in these parameters through beneficial effects on insulin resistance^24^ either via reduction of hepatic lipogenesis^25^ or by increasing urine glucose excretion and thereby caloric loss^26^ or possibly by a direct effect of SGLT2Is on pancreatic alpha-cells.^27^ The small sample size and missing data may explain the lack of significant associations in this study. SGLT2Is have been shown to increase total cholesterol, LDL, and HDL, and decrease TG. The slight increase in LDL appears to be due to large LDL particles which are thought to be less atherogenic. The small dense LDL particles are decreased^28^ which may explain the cardiovascular benefits of these drugs. This study showed a similar increase in LDL and decrease in TG, although the short duration and missing data does not allow for assessment of long-term changes or cardiovascular benefits. Studies suggest that the compliance of statin intake in diabetic patients on SGLT2Is may be affected after observing the beneficial effects of these inhibitors.^29^ Additional clinical trials are needed to evaluate the exact nature of SGLT2Is effects on lipid profiles by comparing patients on statins and not using statins.

Reduction in both liver fat and ALT have been demonstrated with Empagliflozin among T2DM patients with NAFLD, irrespective of glycemic improvement and body weight reduction.^30^ Takase et al found from sub-analysis of 16-weeks, prospective observational study that Ipragliflozin was associated with amelioration of hepatic steatosis by decrease in Fibrosis Index-4 (FIB-4), a surrogate measure for fatty liver^31^, in addition to a reduction in ALT level. A meta-analysis on eight RCTs reported that weight loss of at-least 5% of body weight was associated with a reduction in hepatic steatosis^32^ and hence this variable could have acted as confounder since SGLT2Is also led to weight reduction by decreasing fat mass via substrate utilization of lipids instead of carbohydrates.^33^ Similarly, good glycemic control could have confounded the effects of SGLT2Is as NAFLD is less common in patients with well-controlled blood glucose levels.^34^ GEE for MLR was used in the analysis to address the confounders. Lifestyle modification is a cornerstone of T2DM management and patients are counseled at each follow-up visit. As such, it can be another factor that affects study findings as during this study period, some patients may have had modifications of risk factors between visits which cannot be measured objectively.

Many medications are known to affect liver fat.^35^ Information regarding medications other than SGLT2Is taken by study participants was collected. Specifically, 84% of patients were on Metformin, 6.7% on Liraglutide, 7.5% on TZDs. 10.8% on Vit E and 3.3% on Omega 3. However, the sparse number of patients on these drugs did not allow to take into account these medicines for analysis. Any analysis to observe the true effect of SGLT2Is on the changes in ALT and glycemic parameters by removing subjects on these medications would have reduced the power of our study.

### Limitations:

The retrospective, cohort design increases the risk of information bias. We could not achieve the target sample size that we calculated, hence reducing the power of our study, and increasing the chances of type 2 error. In the designated study period, SGLT2Is were newly available drugs and were prescribed to nearly all patients with uncontrolled diabetes. As such, it was not feasible to select patients not on SGLT2Is as a control arm. We also could not use historical controls (i.e. using patients when the study drug was not available) as the patients may differ in their dietary habits, physical activity level, and follow-up visits in both comparison groups that would have again biased our results since appropriate control group should have similar age, gender, living environments, and health status as the case group.

Another limitation was that only elevated ALT levels were used as the primary outcome measurement for NAFLD. A diagnostic modality, either imaging or Fibro scan, to define or follow NAFLD progression was not used in this retrospective record review as not all patients are advised to undergo these tests by their primary care physicians. As ALT is a biomarker of liver inflammation, it served as a surrogate (proxy indicator) for fatty infiltration that is known to be associated with inflammation. The age range specified in the eligibility criteria (30-60 years) may limit the generalizability of our findings as many studies have included subjects aged up to 70 years and above.

### Recommendations:

This small, retrospective observational study provides important, albeit limited information, for improvement in NAFLD among T2DM with SGLT2Is in Pakistan. Randomized control trials (RCTs) can better compare SGLT2Is with other treatment regimens to demonstrate a reduction in hepatic steatosis in both non-diabetic and diabetic populations and T2DM patients with and without Metabolic Syndrome. Robust studies of SGLT2Is actions on liver fibrosis are needed, using comparatively safer, and non-invasive techniques, such as Fibro scan^36^ and magnetic resonance spectroscopy (MRS)^37^, that can assess liver fat and fibrosis, and have shown to have a good correlation with histologic changes from biopsy. In a cross-sectional study on NAFLD, non-diabetic patients found a positive association of CK18 with serum ALT.^38^ RCTs using T2DM patients having NAFLD can give further evidence of this cost-effective non-invasive approach for improved management of twin comorbidities.

## CONCLUSION

The addition of oral SGLT2Is leads to reduction in mean ALT levels when included in the standard treatment of T2DM patients comorbid with NAFLD. This study shows the beneficial effects of SGLT2-I, a proven approach to glycemic control; with a low adverse event profile in Pakistani population. RCTs are needed to provide further support to consideration of SGLT2Is use when compared with other drugs that may decrease liver fat among these subjects.

Apart from encouraging diet and lifestyle modification at public level, early intervention with SGLT2Is by clinicians may improve metabolic factors as well as hepatic dysfunction and hence, decrease the burden of associated increased morbidity resulting from NAFLD across different diabetic populations.
